# Molecular Imaging of Pulmonary Inflammation in Users of Electronic and Combustible Cigarettes: A Pilot Study

**DOI:** 10.2967/jnumed.122.264529

**Published:** 2023-05

**Authors:** Reagan R. Wetherill, Robert K. Doot, Anthony J. Young, Hsiaoju Lee, Erin K. Schubert, Corinde E. Wiers, Frank T. Leone, Robert H. Mach, Henry R. Kranzler, Jacob G. Dubroff

**Affiliations:** 1 Department of Psychiatry, Perelman School of Medicine, University of Pennsylvania, Philadelphia, Pennsylvania;; 2 Department of Radiology, Perelman School of Medicine, University of Pennsylvania, Philadelphia, Pennsylvania;; 3 Comprehensive Smoking Treatment Program, Penn Lung Center, Philadelphia, Pennsylvania; and; 4 Crescenz VAMC, Philadelphia, Pennsylvania

**Keywords:** electronic cigarettes, cigarettes, PET, ^18^F-NOS, inflammation

## Abstract

Electronic cigarette (EC) use has increased dramatically, particularly among adolescents and young adults, and, like cigarette use, can cause pulmonary inflammation and increase the risk of lung disease. **Methods:** This preliminary study used PET with ^18^F-6-(1/2)(2-fluoro-propyl)-4-methylpyridin-2-amine (^18^F-NOS) to quantify inducible nitric oxide synthase expression to characterize oxidative stress and inflammation in the lungs in vivo in 3 age- and sex-matched groups: 5 EC users, 5 cigarette smokers, and 5 controls who had never smoked or vaped. **Results:** EC users showed greater ^18^F-NOS nondisplaceable binding potential (BP_ND_) than cigarette smokers (*P* = 0.03) and controls (*P* = 0.01), whereas BP_ND_ in cigarette smokers did not differ from that in controls (*P* > 0.1). ^18^F-NOS lung tissue delivery and inducible nitric oxide synthase distribution volume did not significantly differ among groups. Although there were no group differences in peripheral inflammatory biomarker concentrations, ^18^F-NOS BP_ND_ correlated with the proinflammatory cytokine tumor necrosis factor-α concentrations (*r_s_* = 0.87, *P* = 0.05) in EC users. Additionally, when EC users and cigarette smokers were pooled together, number of vaping episodes or cigarettes per day correlated with interleukin-6 levels (*r_s_* = 0.86, *P* = 0.006). **Conclusion:** This is the first PET imaging study to compare lung inflammation between EC and cigarette users in vivo. We found preliminary evidence that EC users have greater pulmonary inflammation than cigarette smokers and controls, with a positive association between pulmonary and peripheral measures of inflammation.

## Visual Abstract

**Figure:**
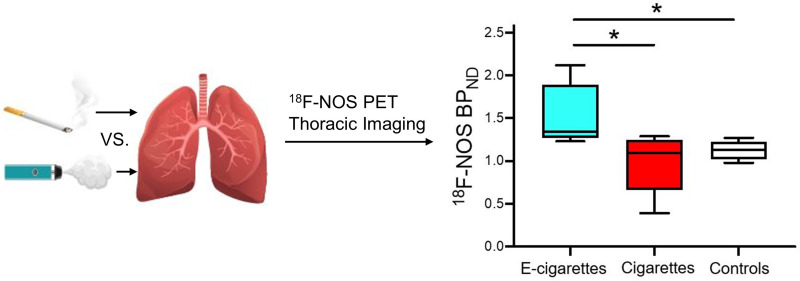

Tobacco use is the world’s leading preventable cause of morbidity and mortality, accounting for more than 8 million deaths annually (1). Although public awareness of smoking-related risks has increased and tobacco smoking has declined, electronic cigarette (EC) use has increased dramatically, particularly among adolescents and young adults (1–3). The increase in EC use is driven partially by the assumption that ECs are safer than conventional cigarettes. Although ECs are often advertised as an alternative smoking cessation tool (4*,*^5^), their long-term effectiveness and safety have not been rigorously evaluated (6*,*^7^). Given the emergence of an epidemic of injuries associated with ECs or vaping products (8), EC use has become a major public health concern, and the adverse pulmonary effects of EC use remain unclear.

ECs deliver nicotine by heating e-liquids (i.e., the liquid used in ECs) containing nicotine in a vegetable glycerin or propylene glycol vehicle with flavorings that are vaporized and inhaled, thus delivering nicotine without combusting tobacco. Although the propylene glycol and vegetable glycerin found in e-liquids are regarded as safe by the U.S. Food and Drug Administration, aerosols from ECs contain tobacco-specific nitrosamines, metals, polycyclic aromatic hydrocarbons, and volatile organic compounds that are known toxicants and carcinogens (9). As with smoking, several EC-related compounds are associated with inflammation, altered innate immune response, oxidative stress, and cytotoxicity (9–11). However, the existing human literature on the pulmonary effects of EC use is limited and comprises mainly studies that use invasive approaches (e.g., induced sputum and bronchoalveolar lavage) that do not adequately assess the impact of EC use on the lungs.

PET imaging has been used to quantify and track inflammatory responses associated with smoking and EC use in vivo without the need for invasive diagnostic studies (12*,*^13^). PET with ^18^F-FDG has been used extensively to detect enhanced glucose metabolic activity of activated immune cells in inflammatory diseases, including pneumonia (14), cystic fibrosis (14), and chronic obstructive pulmonary disease (15). Although associations between ^18^F-FDG quantification and inflammation have been observed, biologic processes, including fibrosis and neoplasia, use glucose and limit the specificity of ^18^F-FDG (16). PET radiotracers targeting the 18-kDa translocator protein, also known as the peripheral benzodiazepine receptor, have also been used to measure pulmonary inflammation (17*,*^18^). These radiotracers were initially considered putative markers of neuroinflammation; however, their specificity for inflammation is limited (19). Thus, recent efforts have focused on imaging specific aspects of immune regulation and response, such as nitric oxide synthase enzymes, with promising results (16*,*^20^).

Nitric oxide plays an important role in immune regulation and is produced by 3 nitric oxide synthase enzymes: neuronal nitric oxide synthase, endothelial nitric oxide synthase, and inducible nitric oxide synthase (iNOS) (21). iNOS is associated with acute and chronic inflammatory diseases, including asthma and chronic obstructive pulmonary disease (22*,*^23^), and is expressed in normal lung epithelium (24). Convergent evidence indicates that iNOS plays a central role in mediating inflammation in smokers of combustible cigarettes, thereby contributing to smoking-related lung diseases. Preclinical models show that chronic exposure to cigarette smoke increases iNOS expression (25), whereas pharmacologic inhibition of iNOS reverses tobacco-induced lung disease (26). Additionally, preclinical research has provided a mechanistic link between iNOS expression in the lung and inflammatory lung diseases (26*,*^27^). These findings strongly support iNOS as a mechanistically relevant target for molecular imaging of lung inflammation and inflammatory lung diseases.

The PET radiotracer ^18^F-6-(1/2)(2-fluoro-propyl)-4-methylpyridin-2-amine (^18^F-NOS) permits the visualization and measurement of in vivo iNOS expression (16*,*^28^). ^18^F-NOS is a radiolabeled version of a reversible iNOS inhibitor with better selectivity than other nitric oxide synthase enzymes (28). ^18^F-NOS has been validated in an animal model of lipopolysaccharide-induced lung injury (29) and was used successfully to image iNOS expression in humans to characterize oxidative stress and inflammation in the heart and lungs (16*,*^28^). This study uses ^18^F-NOS PET lung imaging to quantify differences in iNOS expression among EC users, cigarette smokers, and control subjects who have never smoked or vaped. On the basis of preclinical research showing that exposure to e-liquid vapor and cigarette smoke increases iNOS expression (25*,*^30^), we hypothesized that EC users and cigarette smokers would show greater pulmonary iNOS uptake than would controls. We also assessed blood and plasma inflammatory biomarker concentrations (tumor necrosis factor-α [TNF-α], interleukin-6 [IL-6], and C-reactive protein) and examined their association with ^18^F-NOS PET lung imaging parameters.

## MATERIALS AND METHODS

### Participants

The study protocol was approved by the University of Pennsylvania Institutional Review Board and conducted in compliance with the Health Insurance Portability and Accountability Act under exploratory investigational new-drug number 140,976 for ^18^F-NOS. Participants were recruited via local print media, social media, and previous research studies. Interested individuals completed a brief telephone screen and, if eligible, an in-person intake session during which they provided written informed consent and were screened for eligibility. Twenty-four participants underwent screening, including a physical examination, medical history, routine clinical laboratory tests, and toxicologic urine analysis. Briefly, exclusion criteria included a history or evidence of significant medical disorders, a lifetime *Diagnostic and Statistical Manual of Mental Disorders, Fifth Edition* diagnosis of a psychiatric or substance use disorder (except tobacco use disorder for EC users and cigarette smokers), a positive urine drug screen of drugs of abuse, use of inhaled or oral corticosteroids or antiinflammatory medications, and a past-month history of lung trauma or active lung infection that could impact the uptake of ^18^F-NOS. All female participants had a negative pregnancy test on the scanning day before receiving the radiotracer. Daily for the past 6 mo, EC users had vaped nicotine and cigarette smokers had smoked. The current smoking status was confirmed by carbon monoxide levels greater than 10 parts per million and urine cotinine levels greater than 150 ng/mL. Fifteen age- and sex-matched participants (5 exclusive EC users [mean age, 27 ± 7 y], 5 cigarette smokers [mean age, 35 ± 9 y], and 5 controls [mean age, 28 ± 7 y]), comprising 2 women and 3 men in each group, met the eligibility criteria and completed the study (Supplemental Fig. 1; supplemental materials are available at http://jnm.snmjournals.org).

Before scanning, participants completed the Hospital Depression and Anxiety Scale (31) to assess symptoms of depression and anxiety. EC users completed measures of vaping behavior, including the Penn State Electronic Cigarette Dependence Index (32), and cigarette smokers completed measures of tobacco smoking behavior, including the Fagerström Test for Cigarette Dependence (33). A blood sample was obtained to measure blood or plasma cytokine concentrations (TNF-α, IL-6, and C-reactive protein). Participants underwent dynamic thoracic ^18^F-NOS PET/CT with venous blood sampling.

### Data Acquisition

The PET radiotracer ^18^F-NOS was synthesized as previously described (28). Participants were scanned with an Ingenuity PET/CT scanner (Philips Healthcare), which has a PET spatial resolution of 5 mm in full width at half maximum and an 18-cm axial field of view (34). For each scan, a nuclear medicine physician determined the thoracic field of view that best included the heart and lungs. After a low-dose attenuation-correction CT scan, a 1-h PET dynamic acquisition was started at the time of an intravenous bolus injection of ^18^F-NOS (199 ± 27 MBq) with the following framing schedule: 24 × 5 s, 6 × 10 s, 3 × 20 s, 2 × 30 s, 5 × 60 s, and 10 × 5 min. On the basis of published effective dose estimates of 15.9 μSv/MBq for ^18^F-NOS, 199 MBq delivers an effective dose of 3.16 mSv, with a maximum critical dose to the urinary bladder wall of 19.0 mSv (28). The attenuation-correction CT images were reconstructed into PET images using a previously described list-mode, blob-based ordered-subsets maximum-likelihood expectation-maximization algorithm, including flight-time and physical-data corrections (34). The radiologist who reviewed the images was masked to participant group status, as was the data analyst.

### Metabolite Analysis

Venous blood was sampled at approximately 2, 5, 10, 15, 30, 45, and 60 min after injection to measure radiometabolites. The whole-blood and plasma activity concentrations were counted using a WIZARD^2^ 2480 γ-counter (Perkin Elmer). Acetonitrile-treated plasma supernatant was analyzed in a 1260 Infinity Series (Agilent Technologies) high-performance liquid chromatology system using an Agilent ZORBAX StableBond C18 column via a mobile phase of 73% 0.1 M ammonium formate buffer and 27% methanol. The resulting plasma–to–whole-blood ratio as a function of time was used to convert the image-derived whole-blood input function into a plasma input function. The resulting parent PET radiotracer fraction as a function of time and the plasma input function were inputted for subsequent kinetic analysis.

### Volumes of Interest

**FIGURE 1.**
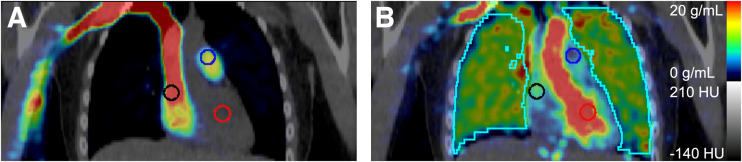
Representative coronal PET/CT images after injecting 207 MBq of ^18^F-NOS, with lung volume of interest (cyan) and 2-cm-diameter spheric blood pool volumes of interest in right atrium (black), pulmonary artery (blue), and left ventricle (red). PET summed uptake is shown from 0 to 15 s after injection (A) and from 37 to 42 s after injection (B). HU = Hounsfield units.

Time–activity curves for the whole blood pool were measured using 1 cm^3^ peak volumes of interest within 2-cm-diameter spheric volumes of interest within the pulmonary artery, as this blood pool is sufficiently large to minimize partial-volume effects and is located immediately before blood enters the lungs (Fig. 1). Lung uptake time–activity curves were extracted from all lung tissue in the PET field of view (Fig. 1).

### Kinetic Analysis

Three models of kinetic analysis were compared for estimating the total volume of distribution (V_T_) from the observed reversible tracer binding: graphical Logan plot (35), 1 tissue compartment, and 2 tissue compartments (2TCs). The 2TC model, with an average whole-lung Akaike information criterion (36*,*^37^) of 184 ± 31, was selected over the 1-tissue-compartment model, with a corresponding Akaike information criterion of 295 ± 24, because the 2TC had the lower, and therefore better, Akaike information criterion score. V_T_ values estimated via the Logan and 2TC models were similar (*R*^2^ = 0.99). As expected, V_T_ values from Logan plots were less biased than when using the 2TC model, with the magnitude of the Logan plot V_T_ bias decreasing with increases in the duration of the PET acquisition. Thus, we used the 2TC model to quantify tracer uptake to avoid having metrics dependent on the PET scan duration.

Two approaches for blood volume fraction (vB) were examined for each model: fixed at 0.15 and floating between 0.05 and 0.3. The floating vB resulted in the least model variability. Kinetic analyses using a 2TC model with a floating lung vB were performed to estimate V_T_, transport into the first tissue compartment (*K*_1_), the distribution volume of the first tissue compartment (*K*_1_/*k*_2_), and nondisplaceable binding potential (BP_ND_) via Pmod image analysis software (version 3.7; PMOD Technologies Ltd.) using the combined lung time–activity curve and PET image-derived plasma input function from the pulmonary artery blood pool (Fig. 1) (38). Kinetic analyses were based on the first 40 min of the PET acquisition to allow a consistent analysis of all participants’ data after 1 participant’s excessive motion resulted in unevaluable PET images after 40 min.

### Statistical Analysis

All statistical tests were 2-sided. Nonparametric Mann–Whitney and Kruskal–Wallis tests were used to assess group differences. Spearman rank-order correlations measured the strength and direction of associations between inflammatory biomarkers, nicotine use behaviors (cigarettes per day for cigarette smokers; vaping episodes per day for EC users), and imaging parameters.

## RESULTS

On average, EC users reported 7 ± 4 vaping episodes/d, with Penn State Electronic Cigarette Dependence Index scores of 6 ± 4, indicating moderate-to-high levels of EC dependence. Cigarette smokers reported smoking 8 ± 4 cigarettes/d, with Fagerström Test for Cigarette Dependence scores of 5 ± 2, indicating moderate levels of cigarette dependence. There were no significant group differences in age, depression and anxiety scores, injected mass radioactivity dose, or plasma-free fraction.

**FIGURE 2.**
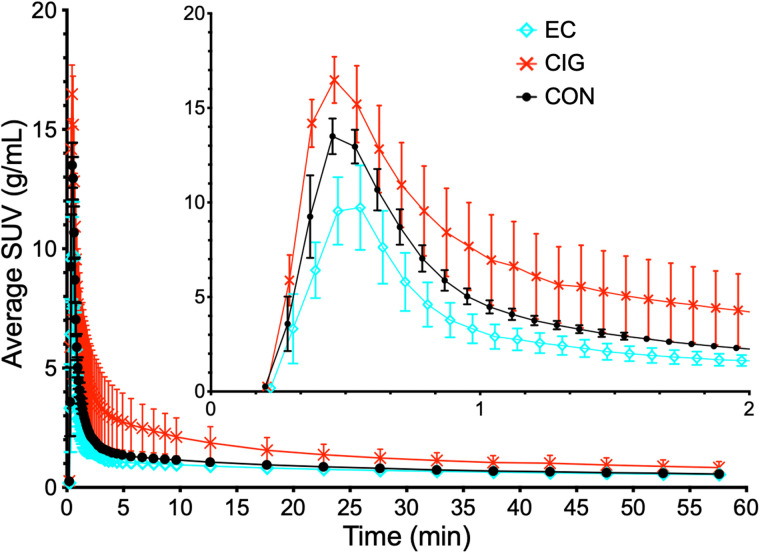
Average lung ^18^F-NOS uptake for each group as function of time, with SD error bars. CIG = cigarette smokers; CON = controls.

Selection of the pulmonary artery to measure the blood input function is supported by the example PET/CT images in Figure 1, where the distribution of ^18^F-NOS before entry into the lungs and then the left ventricle indicates that ^18^F-NOS enters the right atrium, followed by the pulmonary artery. Figure 2 shows average lung ^18^F-NOS uptake for all participants as a function of time. Table 1 presents kinetic analysis results, where the average estimate of 0.15 ± 0.02 for lung vB is consistent with the reported normal lung vB range of 0.14–0.19 from ^18^F-FDG PET/CT scans (39).

**TABLE 1.** Kinetic Analysis Results

Patient no.	Sex	V_T_	*K*_1_	*K*_1_/*k*_2_	BP_ND_	vB
EC-07	F	1.17	1.62	0.51	1.31	0.15
EC-10	M	0.63	1.42	0.28	1.23	0.16
EC-13	F	0.99	1.58	0.42	1.34	0.18
EC-20	M	1.20	2.67	0.39	2.12	0.18
EC-23	M	0.83	1.26	0.31	1.66	0.13
CIG-12	M	1.10	2.71	0.57	0.93	0.14
CIG-14	M	4.74	2.29	3.42	0.39	0.14
CIG-17	M	1.14	3.24	0.52	1.21	0.16
CIG-22	F	1.45	2.70	0.66	1.29	0.11
CIG-24	F	1.06	1.62	0.51	1.09	0.15
CON-01	F	1.15	1.95	0.58	0.98	0.18
CON-03	M	1.04	1.85	0.48	1.17	0.15
CON-05	M	0.91	1.37	0.40	1.27	0.15
CON-06	F	1.53	3.19	0.74	1.07	0.17
CON-09	M	1.18	1.62	0.56	1.13	0.13
ECs		0.97 ± 0.24	1.71 ± 0.56	0.38 ± 0.09*	1.53 ± 0.37*	0.16 ± 0.02
CIGs		1.90 ± 1.60	2.51 ± 0.60	1.13 ± 1.28	0.98 ± 0.36	0.14 ± 0.02
CONs		1.16 ± 0.23	1.99 ± 0.70	0.55 ± 0.13	1.12 ± 0.11	0.16 ± 0.02
All		1.34 ± 0.97	2.07 ± 0.67	0.69 ± 0.77	1.21 ± 0.37	0.15 ± 0.02
*P*		0.36	0.09	0.03	0.02	0.31

* *P* < 0.05 on comparison between ECs and NUs.

EC = EC user; CIG = combustible cigarette user; CON = control.

Data are mean values and mean ± SD. *P* values are from Kruskal–Wallis test comparing 3 groups.

**FIGURE 3.**
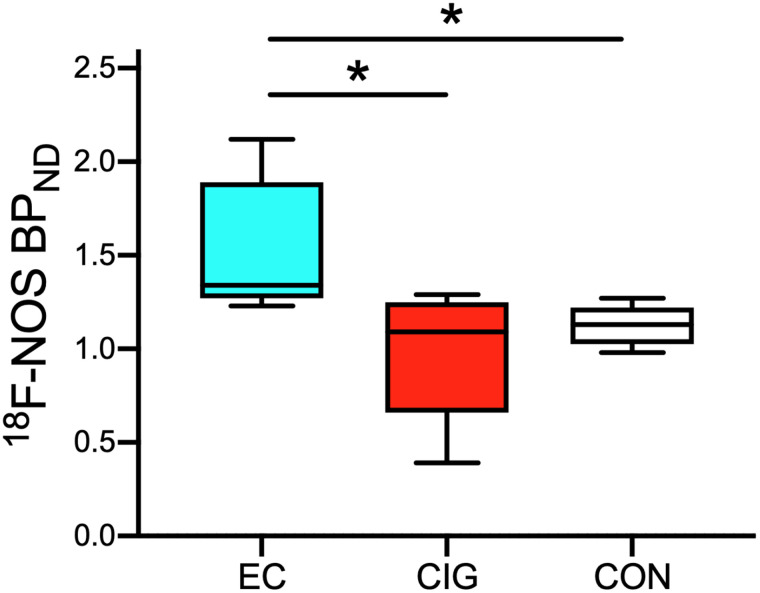
Box plot of ^18^F-NOS BP_ND_ by group. EC users show higher ^18^F-NOS BP_ND_ than controls (*P* = 0.01) and cigarette smokers (*P* = 0.03). *P < 0.05. CIG = cigarette smokers; CON = controls.

^18^F-NOS BP_ND_ values differed significantly among groups (H(2) = 7.50, *P* = 0.02; Fig. 3). Post hoc comparisons revealed that EC users had higher BP_ND_ values than cigarette smokers (*P* = 0.03) and controls (*P* = 0.01). ^18^F-NOS V_T_ and *K*_1_ values did not differ among groups (*P* > 0.09).

Peripheral inflammatory biomarker concentrations did not differ among groups (*P* > 0.16). Spearman rank-order correlations examined the associations between daily smoking or vaping behavior, inflammatory biomarker concentrations, and imaging parameters. There was a positive correlation among EC users between ^18^F-NOS BP_ND_ and TNF-α concentration (*r_s_* = 0.87, *P* = 0.05; Supplemental Fig. 2). Among EC users and cigarette smokers, cigarettes per day and vaping episodes per day correlated with IL-6 levels (*r_s_* = 0.89, *P* = 0.001; Supplemental Fig. 3). No other correlations were statistically significant.

## DISCUSSION

EC use has increased dramatically, particularly among adolescents and young adults. Consequently, well-controlled studies are urgently needed to examine and compare the effects of EC use and cigarette smoking. The existing literature comprises mainly cell culture studies or in vivo animal studies. A few studies examine the effects of EC use on the human lung based on invasive approaches that do not assess the global burden of EC use on the lungs. This preliminary study addressed these gaps using noninvasive, ^18^F-NOS PET lung imaging to quantify and compare lung inflammation in exclusive EC users, exclusive cigarette smokers, and controls. Our preliminary ^18^F-NOS PET findings show that EC users, cigarette smokers, and controls have similar delivery of ^18^F-NOS to the lung tissue and similar iNOS availability. However, ^18^F-NOS BP_ND_ was significantly higher in the EC group than in cigarette smokers and controls. Moreover, ^18^F-NOS BP_ND_ in EC users was associated with the proinflammatory cytokine TNF-α. Number of cigarettes and vaping episodes per day correlated with IL-6 levels. To our knowledge, this was the first PET lung imaging study demonstrating that EC users show a unique PET lung phenotype associated with known inflammatory biomarkers.

Although we did not see the expected increase in ^18^F-NOS uptake in cigarette smokers, our findings are consistent with recent work that used bronchoscopy to isolate alveolar macrophages from bronchoalveolar lavage samples in smokers, EC users, and never-smokers and found that EC users showed greater iNOS expression in alveolar macrophages than did smokers or never-smokers (40). Animal and human studies have shown that iNOS expression is induced in most cell types on exposure to inflammatory stimuli (41) and is associated with increased pulmonary nitric oxide (42). Nitric oxide mediates neutrophil and macrophage actions that are thought to contribute to pulmonary oxidant stress and acute lung injury (43). Thus, our findings suggest that EC use may alter pulmonary oxidative stress responses and predispose them to acute lung injury.

Although groups showed similar levels of inflammatory biomarkers, EC users showed positive associations between ^18^F-NOS PET imaging parameters and TNF-α concentration. TNF-α is a proinflammatory cytokine produced by macrophages and secreted by neutrophil granulocytes at sites of injury (44) and is involved in the inflammatory cascade of acute lung injury (45). Indeed, studies have shown that proinflammatory cytokines induce iNOS expression in human alveolar cells in response to exposure to fine particulate matter (46). As such, our findings provide additional evidence of the altered immune responses in the lungs of EC users.

Several limitations of this study should be considered. First, we did not account for vaping topography (i.e., how an EC is used, including puff duration, puff volume, and EC device and power settings). These factors are important in differential exposure to nicotine and toxicants among EC users (47). Although we used individually measured PET radiotracer parent fractions as a function of time to correct for the presence of radiolabeled metabolites in the blood, we could not separate lung ^18^F-NOS uptake due to binding of parent ^18^F-NOS from any binding of radiolabeled metabolites. Huang et al. asserted that “because of [the metabolite’s] polarity, this metabolite is most likely excluded by the lung endothelium from entering the lung parenchyma” (16). Impacts of any lung binding of radiolabeled metabolites on estimates of ^18^F-NOS BP_ND_ will likely be inversely related to the validity of the assumption that polar ^18^F-NOS metabolites cannot penetrate lung endothelium. To date, no studies have provided information on the reproducibility of the ^18^F-NOS PET assay; however, previous research showed consistent findings in left- and right-lung ^18^F-NOS parameters (16). In addition, because the sample sizes were small, additional larger studies are needed to replicate these findings and provide greater statistical power for secondary analyses.

## CONCLUSION

Using rigorous quantitative methods and a global technique to examine pulmonary oxidative stress, we found evidence that EC use causes a unique inflammatory response in the lungs, reflected by PET measures of iNOS expression and correlations with inflammatory biomarker concentrations. Future work is needed to elucidate the effect of EC use on respiratory health, especially the effects of chronic EC use.

## DISCLOSURE

The study was supported by the National Heart, Lung, and Brain Institute (R21HL144673), the National Institute on Drug Abuse (P30DA046345), the National Center for Advancing Translational Sciences of the National Institutes of Health (UL1TR001878), and in part by the Institute for Translational Medicine and Therapeutics’ (ITMAT) Transdisciplinary Program in Translational Medicine and Therapeutics. Henry Kranzler is a member of advisory boards for Dicerna Pharmaceuticals, Sophrosyne Pharmaceuticals, and Enthion Pharmaceuticals; a consultant to Sobrera Pharmaceuticals; recipient of grant funds and medication supplies from Alkermes for an investigator-initiated study; a member of the American Society of Clinical Psychopharmacology’s Alcohol Clinical Trials Initiative, which was supported by Alkermes, Dicerna, Ethypharm, Lundbeck, Mitsubishi, and Otsuka; and a holder of U.S. patent 10,900,082, titled: “Genotype-Guided Dosing of Opioid Agonists,” issued January 26, 2021. Jacob Dubroff, Robert Doot, and Robert Mach have received support from the Michael J. Fox Foundation. No other potential conflict of interest relevant to this article was reported.

## Notes

KEY POINTS **QUESTION:** What are the effects of EC use on pulmonary inflammation compared with combustible cigarette use and no use of cigarettes or vaping, as measured with ^18^F-NOS PET imaging? **PERTINENT FINDINGS:** In this preliminary PET imaging study, EC users showed greater ^18^F-NOS BP_ND_ than cigarette smokers and controls. ^18^F-NOS BP_ND_ significantly correlated with the proinflammatory cytokine TNF-α in EC users. Additionally, when EC users and cigarette smokers were pooled together, vaping episodes and cigarettes per day correlated with IL-6 levels. **IMPLICATIONS FOR PATIENT CARE:** Preliminary data indicate that EC users show a unique PET lung imaging phenotype associated with known proinflammatory cytokines, suggesting that EC use may increase pulmonary inflammation.
